# *In Vivo* Toxicity of Intravenously Administered Silica and Silicon Nanoparticles

**DOI:** 10.3390/ma5101873

**Published:** 2012-10-16

**Authors:** Sergey Ivanov, Sergey Zhuravsky, Galina Yukina, Vladimir Tomson, Dmitry Korolev, Michael Galagudza

**Affiliations:** 1 Institute of Experimental Medicine, V.A. Almazov Federal Heart, Blood and Endocrinology Centre, Akkuratova str., 2, 197341St-Petersburg, Russian Federation; E-Mails: serjivanov84@gmail.com (S.I.); s.jour@mail.ru (S.Z.); dimon@cardioprotect.spb.ru (D.K.); 2 Department of Pathophysiology, I.P. Pavlov Federal Medical University, Lev Tolstoy str., 6/8, 197022 St-Petersburg, Russian Federation; 3 Laboratory of Hearing and Speech, Core Research Centre, I.P. Pavlov Federal Medical University, Lev Tolstoy str., 6/8, 197022 St-Petersburg, Russian Federation; 4 Division of Pathomorphology, Core Research Centre, I.P. Pavlov Federal Medical University, Lev Tolstoy str., 6/8, 197022 St-Petersburg, Russian Federation; E-Mails: pipson@inbox.ru (G.Y.); tomsonvv@spb-gmu.ru (V.T.); 5 Laboratory of Biophysics of Circulation, Institute of Cardiovascular Diseases, I.P. Pavlov Federal Medical University, Lev Tolstoy str., 6/8, 197022 St-Petersburg, Russian Federation

**Keywords:** silicon nanoparticles, silica nanoparticles, *in vivo* toxicity

## Abstract

Both silicon and silica nanoparticles (SiNPs and SiO_2_NPs, respectively) are currently considered to be promising carriers for targeted drug delivery. However, the available data on their *in vivo* toxicity are limited. The present study was aimed at investigation of SiNP and SiO_2_NP (mean diameter 10 and 13 nm, respectively) toxicity using both morphological and functional criteria. Hematological and biochemical parameters were assessed in Sprague-Dawley rats 5, 21 and 60 days after administration of NPs. Inner ear function was determined using otoacoustic emission testing at 21 and 60 days after infusion of NPs. Furthermore, the histological structure of liver, spleen and kidney samples was analyzed. Intravenous infusion of SiNPs or SiO_2_NPs (7 mg/kg) was not associated with significant changes in hemodynamic parameters. Hearing function remained unchanged over the entire observation period. Both inter- and intragroup changes in blood counts and biochemical markers were non-significant. Histological findings included the appearance of foreign body-type granulomas in the liver and spleen as well as microgranulation in the liver after administration of NPs. The number of granulomas was significantly lower after administration of SiNPs compared with SiO_2_NPs. In conclusion, both tested types of NPs are relatively biocompatible nanomaterials, at least when considering acute toxicity.

## 1. Introduction

Targeted drug delivery (TDD) to an area of interest offers the advantages of decreased drug toxicity and improved therapeutic efficacy because of the selective accumulation of the therapeutic agent within the diseased tissue [1]. While most of the work on TDD has focused on tumor therapy (for review, see [2] and references therein), it has more recently been applied to the treatment of ischemic heart disease [3], and inflammatory [4] and autoimmune [5] diseases. The concept of TDD is based on the fact that intravenously administered drug-loaded nanocarriers can accumulate in the affected tissue, a process followed by local drug release. A number of both organic and inorganic nano-sized carriers, including liposomes, polymeric micelles, dendrimers, drug-polymer conjugates and metal nanoparticles (NPs) have been shown to provide targeted delivery of pharmaceuticals or genes to specific tissues [6,7]. The major concerns associated with the use of nanoparticulate carriers for TDD are their toxicity and biodegradability. Ideally, a nanocarrier should have the following characteristics: sufficient drug loading capacity, high potential for accumulation in the diseased tissue, controlled drug release kinetics, biological inertness and complete biodegradation.

Among inorganic nanocarriers, both silicon- and silica-based NPs (SiNPs and SiO_2_NPs, respectively) have received considerable interest in the past few years [8,9]. As far as SiO_2_NPs are concerned, the existing data on their toxicity remain controversial [10]. Several *in vitro* studies have convincingly demonstrated that mesoporous [11] and colloidal SiO_2_NPs [12] do not affect cell viability or plasma membrane integrity at concentrations adequate for potential pharmacological applications. However, other studies provided evidence of SiO_2_NP-mediated cytotoxicity, which was dose-, time- and size-dependent [13,14]. The data on *in vivo* toxicity of SiO_2_NPs are even more contradictory. In particular, Kumar *et al*. [15] showed a complete clearance of organically modified, 20–25 nm SiO_2_NPs from nude mice. This clearance occurred via hepatobiliary excretion within 15 days after a single intravenous infusion, with no sign of organ toxicity. In contrast, Xie *et al*. [16], in a partially analogous experimental model, demonstrated extensive liver injury *(i.e.,* hepatocyte necrosis and mononuclear infiltration) accompanied by SiO_2_NP retention in the reticulo-endothelial system (RES) for over 30 days. A similar hepatotoxic effect after either single or repeated SiO_2_NP administration was also reported by others [17,18]. The differences in the hepatotoxic effects, apart from other factors, might be accounted for by the distinct characteristics of particle size [19], surface charge [20], or both. At present, there is some evidence that porous SiNPs are more readily biodegradable than SiO_2_NPs. For instance, Park *et al*. [8] observed complete clearance of porous 126 nm SiNPs at four weeks after intravenous administration in mice. However, to our knowledge, the *in vivo* toxicity of colloidal SiNPs has not been previously described in the literature.

The present study was aimed at comparative assessment of the *in vivo* toxicity of SiNPs and SiO_2_NPs in a rat model. The acute effects of intravenous infusions of SiNPs and SiO_2_NPs on hemodynamic variables were studied first. The effects of intravenously administered SiNPs and SiO_2_NPs on body weight, hematological parameters, and biochemical serum markers were also examined at the 5th, 21st, and 60th day after exposure. In addition, inner ear function and histology of the liver, spleen, and kidney were investigated after administration of either SiNPs or SiO_2_NPs.

## 2. Results and Discussion

### 2.1. Characterization of SiNPs and SiO_2_NPs

**Figure 1:**
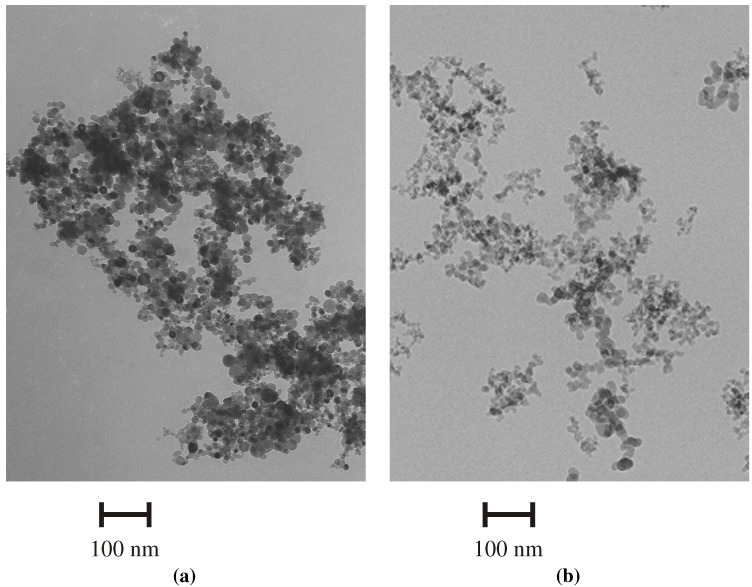
Electron micrographs of (**a**) SiNPs; and (**b**) SiO_2_NPs.

**Figure 2:**
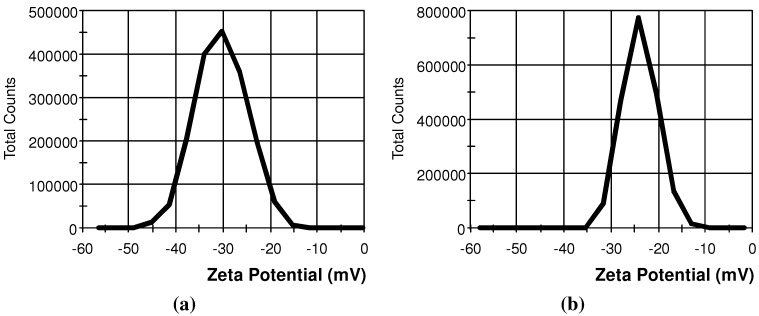
Distribution of zeta potential for (**a**) SiNPs; and (**b**) SiO_2_NPs.

Transmission electron microscopy (TEM) images of SiNPs and SiO_2_NPs are presented in Figure 1. According to TEM, the mean particle diameter was 10 ± 5 and 13 ± 2 nm for SiNPs and SiO_2_NPs, respectively. The distribution of zeta potential for both types of NPs is shown in Figure 2. Numerical values of zeta potential, conductivity of nanoparticle (NP) suspensions, hydrodynamic diameters of NP agglomerates and specific surface areas are shown in Table 1.

**Table 1:** Physicochemical characteristics of SiNPs and SiO_2_NPs. Values are means ± SD.

Characteristics	SiNPs	SiO_2_NPs
Zeta potential, mV	23.8 ± 3.74	30.4 ± 5.45
Conductivity, mS/cm	0.0155 ± 0.00082	0.00574 ± 0.00024
Hydrodynamic diameter, nm	150 ± 27	240 ± 114
BET surface area, m^2^/g	103 ± 12	175 ± 24

### 2.2. Hemodynamic Parameters

The values of MAP and HR were not different either within or between groups (Table 2), indicative of a lack of acute toxicity of either SiNPs or SiO_2_NPs at the selected dose.

**Table 2:** The effect of intravenous administration of silicon and silica nanoparticles on hemodynamic parameters in rats. HR—heart rate; MAP—mean arterial pressure; SiNP—silicon nanoparticles; SiO_2_NP—silica nanoparticles. Values are means ± SD.

Please add the title		Control (n = 6)		SiNPs (n = 7)		SiO_2_NPs (n = 6)	
		HR (bpm)	MAP (mm Hg)	HR (bpm)	MAP (mm Hg)	HR (bpm)	MAP (mm Hg)
I	2 min before infusion	378 ± 36	106 ± 18	389 ± 37	115 ± 24	368 ± 33	110 ± 13
	End of infusion	375 ± 32	108 ± 12	382 ± 28	111 ± 20	371 ± 34	109 ± 17
	10 min after infusion	389 ± 42	112 ± 15	379 ± 38	114 ± 18	364 ± 39	114 ± 21
II	2 min before infusion	368 ± 37	111 ± 16	374 ± 36	108 ± 21	382 ± 31	118 ± 23
	End of infusion	354 ± 38	109 ± 22	365 ± 34	112 ± 25	375 ± 30	119 ± 17
	10 min after infusion	343 ± 33	105 ± 14	376 ± 32	117 ± 28	360 ± 27	115 ± 12
III	2 min before infusion	346 ± 39	118 ± 16	374 ± 36	115 ± 23	361 ± 37	117 ± 10
	End of infusion	351 ± 36	116 ± 19	378 ± 25	109 ± 17	365 ± 32	112 ± 18
	10 min after infusion	342 ± 35	114 ± 17	380 ± 48	113 ± 15	357 ± 27	108 ± 24

### 2.3. Animal Body Weight

**Figure 3:**
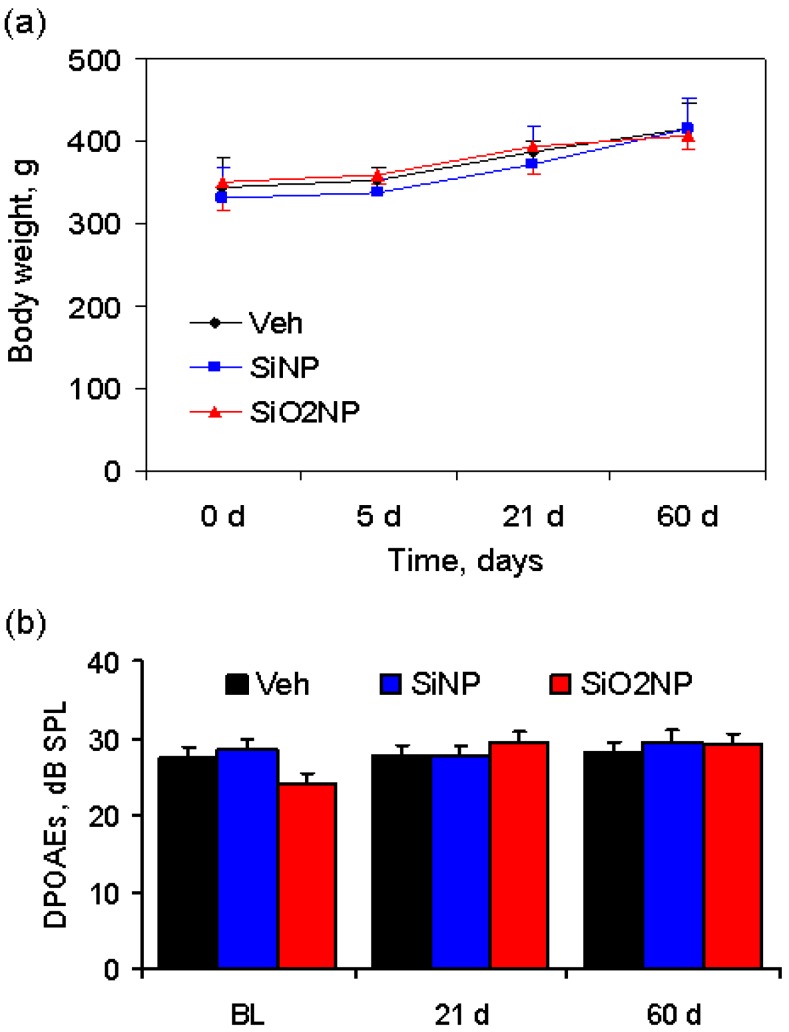
(**a**) Body weight of rats following infusion of vehicle, SiNPs or SiO_2_NPs at a dose of 7 mg/kg. There is no statistically significant difference in body weight between the groups over a period of 60 days; (**b**) Distortion product otoacoustic emission (DPOAE) amplitude measured at the following time points: 24 h prior to treatment, 21 and 60 days after administration of vehicle, SiNPs or SiO_2_NPs. BL—baseline level.

Body weight in vehicle-, SiNP- and SiO_2_NP-treated animals recorded over a period of 60 days is shown in Figure 3(a). During the observation period, the body weight of the rats, injected with either SiNPs or SiO_2_NPs at a dose of 7 mg/kg, increased significantly in a pattern similar to control rats, indicating that the rats continued to mature without any significant toxic effects.

### 2.4. Inner Ear Function

The values of DPOAEs registered at baseline and at the 21st and 60th day after treatment are presented in Figure 3b. The DPOAE amplitude remained unchanged after administration of NPs, suggesting that both types of nanocarriers do not exhibit ototoxicity.

### 2.5. Hematological Parameters

**Figure 4:**
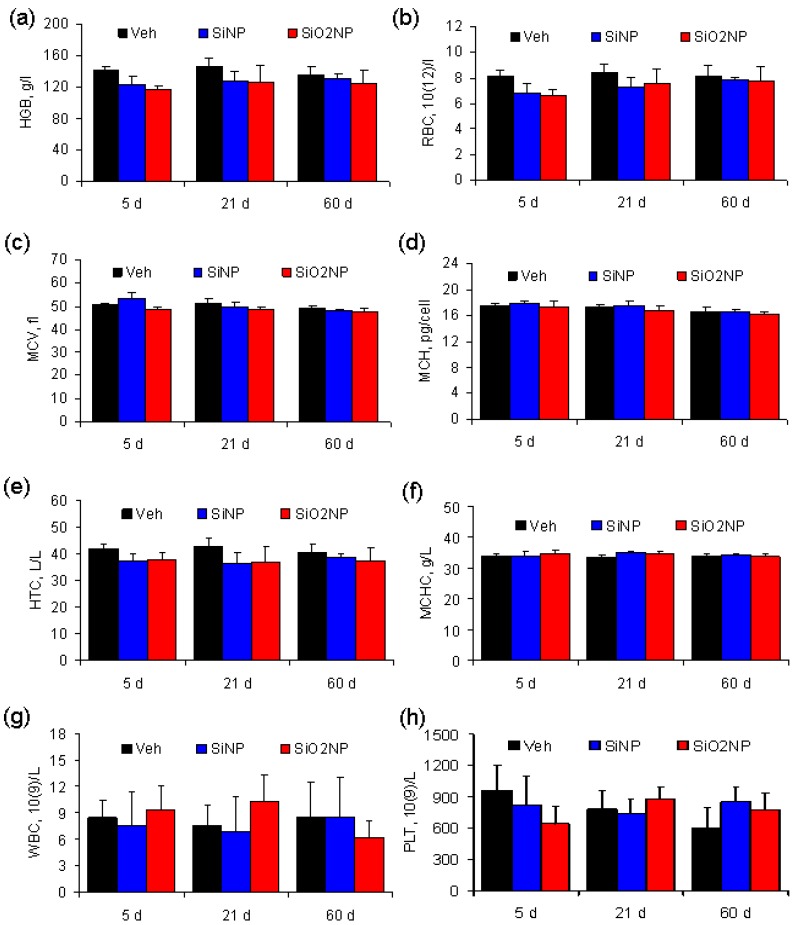
Blood parameters in Sprague-Dawley rats treated with vehicle, SiNPs or SiO_2_NPs at a dose of 7 mg/kg. The results show mean values and SD of (**a**) hemoglobin (HGB); (**b**) red blood cells (RBC); (**c**) mean corpuscular volume (MCV); (**d**) mean corpuscular hemoglobin (MCH); (**e**) hematocrit (HCT); (**f**) mean corpuscular hemoglobin concentration (MCHC); (**g**) white blood cells (WBC); and (**h**) platelets (PLT).

Major hematological parameters measured on the 5th, 21st, and 60th days after treatment are presented in Figure 4. These parameters were not different either within or between groups.

### 2.6. Biochemical Serum Markers

**Figure 5:**
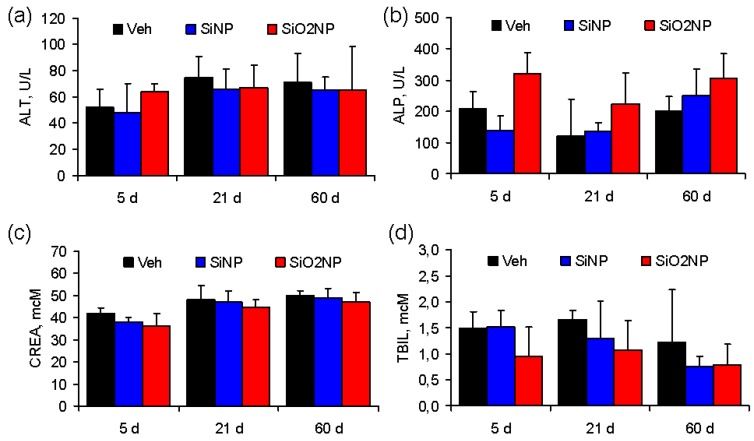
Biochemical serum markers in Sprague-Dawley rats treated with vehicle, SiNPs, or SiO_2_NPs at a dose of 7 mg/kg. The results show mean values and standard deviations of (**a**) alanine transaminase (ALT); (**b**) alkaline phosphatase (ALP); (**c**) creatinine (CREA); and (**d**) total bilirubin (TBIL).

Figure 5 shows the changes in biochemical parameters in the serum of rats treated with vehicle, SiNPs or SiO_2_NPs. Serum ALP activity tended to be higher in the SiO_2_NP-treated rats at 5 and 60 days post-infusion, although the difference was not statistically significant (Figure 5b). Other biochemical markers were not different between the groups.

### 2.7. Histopathological Examination

**Figure 6:**
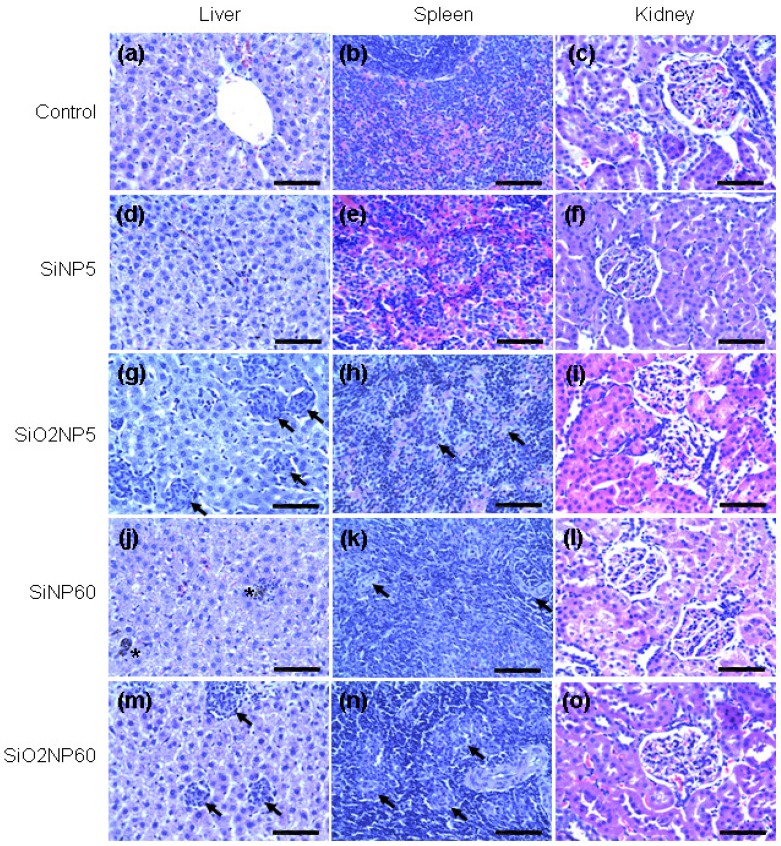
Histological evaluation of organs from rats treated with SiNPs or SiO_2_NPs. Liver, spleen, and kidney samples were collected at 5 and 60 days after intravenous administration of SiNPs or SiO_2_NPs at a dose of 7 mg/kg and fixed with paraformaldehyde, followed by staining with hematoxylin & eosin. (**a–c**) Vehicle-treated animals (controls); (**d–f**) 5 days after SiNP treatment (SiNP5); (**g–i**) 5 days after SiO_2_NP treatment (SiO_2_NP5); (**j–l**) 60 days after SiNP treatment (SiNP60); and (**m–o**) 60 days after SiO_2_NP treatment (SiO_2_NP60). The arrows indicate granulomas in the liver and spleen. The asterisks indicate microgranulation in the liver. The tissue sections were observed under a microscope at 400×. The scale bar is 25 μm for all images. The pictures are representative of at least 4 independent sections.

Representative histological sections of the liver, spleen, and kidney at the 5th and 60th days after treatment are shown in Figure 6. In the liver, multiple foreign body-type granulomas and mononuclear infiltrates were identified in SiO_2_NP-treated animals starting from the 5th day post-infusion (Figure 6g,m). Notably, granulomas were not observed in the liver of SiNP-treated animals at this time point (Figure 6d). However, small, dense granulomas as well as microgranulation of hepatocytes appeared in the liver of SiNP-treated animals at the 21st and 60th days (Figure 6j). The surrounding hepatocytes appeared moderately dystrophic. Starting from the fifth day post-infusion, inflammatory infiltrates consisting of both mononuclear cells and granulomas were also observed in the spleen in both SiNP- and SiO_2_NP-treated animals (Figure 6h,k,n). The mononuclear inflammatory infiltrates in the spleen were more variable in both size and shape in comparison to those seen in the liver. There were no apparent histological abnormalities in the kidney samples related to treatment with either type of NP (Figure 6f,I,l,o).

### 2.8. Morphometric Analysis of Granulomas

**Figure 7:**
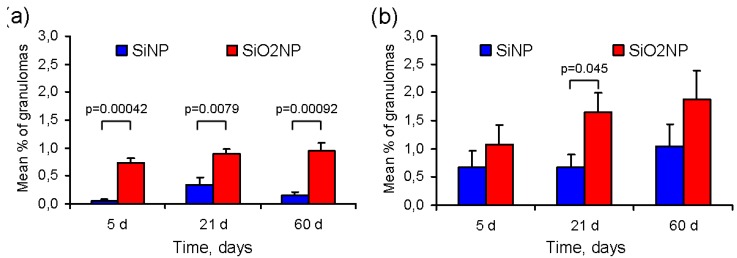
The mean percentage of granulomas in the (**a**) liver; and (**b**) spleen at 5, 21, and 60 days after intravenous infusion of silicon and silica nanoparticles (SiNPs and SiO_2_NPs, respectively). The results are shown as the mean percentage of granuloma area ± SD of six different sections from three different rats (two sections per rat).

The mean percentage of granulomas in the liver and spleen sections is presented in Figure 7. The amount of granulomas in the liver samples was significantly lower in SiNP-treated rats as compared with SiO_2_NP-treated rats at any time point after treatment (Figure 7a). In addition, the density of granulomas in the spleen at the 21st, but not at the 5th or 60th days post-infusion, was also higher in SiO_2_NP-treated rats than in those exposed to SiNPs (Figure 7b). It should be noted that the number of granulomas, at least in the spleen, tended to increase over time in both groups.

The exact mechanisms of biodegradation and clearance of SiNPs and SiO_2_NPs are yet to be determined. It is postulated that gradual biodegradation of both materials results in the formation of water-soluble salts of silicic acid, which are excreted by the kidney. Hepatobiliary excretion may also, at least in part, contribute to clearance of SiO_2_NPs from the body [19]. At present, there is some evidence that hydrogenated, porous SiNPs are more readily biodegradable than SiO_2_NPs. This is thought to be due to the formation of a back-bonded oxygen shell (Si-O-Si bonds), resulting in the rapid layer-by-layer NP dissolution in biological fluids [21]. For instance, porous SiNPs were shown to have a half-life in the blood of approximately only 10 min [8], requiring either thermal oxidation or thermal hydrocarbonization to increase particle half-life and ensure effective biological applications, such as *in vivo* drug delivery. Although the plasma concentration of silicon was not measured in the present study, our findings on the persistence of microgranulation in the liver up to the 60^th^ day after administration of SiNPs suggest that the rate of biodegradation for colloidal SiNPs might be lower than that of porous SiNPs.

In the present study, multiple foreign body-type granulomas were identified after NP administration in RES organs, specifically, in the liver and spleen. Of note, the amount of granulomas was significantly lower in the animals treated with SiNPs compared with those treated with SiO_2_NPs. It is known that exogenously administered NPs are rapidly labeled by certain plasma proteins, a process that is usually referred to as opsonization [22]. The opsonization rate might be decreased if the particle surface is covered by a biocompatible coating, e.g., polyethylene glycol (PEG) [22]. In the present study, the NPs were not PEGylated in order to determine the potential toxic effects of NPs on RES organs.

Opsonized NPs are recognized by scavenger receptors localized on the membrane of tissue resident macrophages, such as Kupffer cells and splenic macrophages [16]. This is followed by the endocytosis of NPs and their internalization into macrophages. The rate of SiO_2_NPs internalization has been shown to depend on both particle size [16] and method of functionalization [23]. The lysosomal enzymes cannot digest inorganic material, which leads to the accumulation of NPs in macrophages. Activated macrophages secrete proinflammatory cytokines, such as interleukin-1, interleukin-6, and tumor necrosis factor α. The macrophage-derived inflammatory cytokines have two major effects: (1) expression of adhesion molecules on endothelial cells for extravasation of monocytes and lymphocytes; and (2) stimulation of targeted migration of mononuclear cells to the area of inflammation (chemotaxis). Thus, additional mononuclear phagocytes are recruited to the tissue from the intravascular space. Both resident and recruited macrophages and lymphocytes are organized into a specific association of inflammatory cells, or granuloma. Interestingly, the extensive formation of granulomas in the liver and spleen was not associated with any appreciable changes in biochemical serum markers in our experiments. This fact suggests that, despite the presence of multiple granulomas, liver function remained unaltered.

Several factors are thought to have a profound impact on the development of nanoparticle-mediated toxicity. One of the key factors is NP diameter, which, at least for colloidal NPs, inversely correlates with the surface area. It has been shown that, in general, smaller NPs with a greater surface area are more toxic both *in vitro* and *in vivo* [10]. With this in mind, we have selected for analysis the smallest SiNPs and SiO_2_NPs of comparable diameter. Other factors include NP dose and treatment regimen. Obviously, higher doses of NPs and/or their repeated administration are associated with higher probability of toxicity. In this work, we studied the effects of single intravenous administration of NPs at a minimal dose required for potential drug delivery applications. The dose of NPs was selected on the basis of our recent work showing that SiNPs at a single dose of 7 mg/kg have adequate loading capacity to adsorb a sufficient amount of adenosine to limit infarct size with greater efficacy than an equivalent dose of free adenosine [3].

Along with the classical indices of toxicity, such as blood counts and histology, the effects of SiNPs and SiO_2_NPs on hearing function were also tested in this study. Although the possibility of inner ear targeting has not been considered before, one cannot exclude that this approach might be effective in treating hearing dysfunction including acute and progressive sensorineural hearing loss as well as presbycusis. Current treatment options include systemic corticosteroid treatment in acute sensorineural hearing loss and systemic administration of otoprotective and metabolic drugs in progressive sensorineural hearing loss and presbycusis. Targeted delivery of the drugs to the inner ear might contribute to attenuation of side effects and increased efficacy. It is conceivable that drug-loaded NPs may cross the blood-labyrinth barrier by means of uptake by inner ear supporting epithelial cells with non-specific phagocytotic activity [24]. Therefore, we were interested to see whether silicon-based NPs affect the function of the inner ear. The lack of significant changes in DPOAEs after administration of SiNPs or SiO_2_NPs could be interpreted as additional evidence for the biocompatibility of the tested nanomaterials.

*In vivo* toxicity of SiO_2_NPs after intravenous administration was previously studied by several groups; Xie *et al*. [16] reported intracellular persistence of 20 and 80 nm SiO_2_NPs in the lungs, liver, and spleen for over 30 days after administration in mice at a dose of 10 mg/kg. The amount of 20 nm SiO_2_NPs in the liver and spleen was higher than that of the 80 nm NPs. Moreover, both types of SiO_2_NPs caused periportal mononuclear infiltration in the liver, as well as hepatocyte necrosis. In a more recent study, the same group demonstrated that systemically administered SiO_2_NPs accumulated mainly in the liver and in the white pulp of the spleen [25]. The NPs underwent both urinary and hepatobiliary excretion from the organism, with the former being more efficient. Nishimory *et al*. [17] compared the effects of 70, 300, and 1000 nm SiO_2_NPs on liver histology and function in mice. SiO_2_NPs (70 nm) caused liver injury at a dose of 30 mg/kg, while 300 and 1000 nm SiO_2_NPs were found to be non-toxic even at 100 mg/kg. Repeated intravenous administration of 70 nm SiO_2_NPs for 4 weeks resulted in hepatic microgranulation and, at a later stage, liver fibrosis. It was shown in mice that organically modified 20–25 nm SiO_2_NPs were completely eliminated from the organism within 15 days after intravenous infusion at a relatively low dose of 2 mg/kg [15]. The LD_50_ of mesoporous hollow SiO_2_NPs was found to be greater than 1000 mg/kg in mice [26]. No histopathological findings in the liver, spleen, lung, or kidney were observed in mice that received mesoporous hollow SiO_2_NPs at single doses ranging from 40 to 160 mg/kg. At the same time, lymphocytic infiltration, microgranulation, and degenerative necrosis of hepatocytes were observed in the liver when hollow SiO_2_NPs were administered at 500 or 1280 mg/kg. Cho *et al*. [19] analyzed tissue distribution and excretion of 50, 100 and 200 nm SiO_2_NPs given to mice at a dose of 50 mg/kg. Surprisingly, the cellular uptake of SiO_2_NPs increased with their size. Particles of all sizes were excreted via urine and bile; the most efficient urinary clearance was observed for 50 nm SiO_2_NPs. The NPs persisted in liver and splenic macrophages for four weeks post-injection. To summarize, most of the *in vivo* studies demonstrated the accumulation and persistence of SiO_2_NPs in macrophages of the liver and spleen, which was associated with a variable degree of inflammatory response. The determinants of the observed toxicity are complex and include particle size, surface area, dose, and treatment regimen. Our results generally confirmed the above findings. Single intravenous administration of 11–15 nm SiO_2_NPs to rats at a dose of 7 mg/kg resulted in granuloma formation and mononuclear infiltration in the liver and spleen that persisted for 60 days post-injection.

The present study has several methodological limitations. First, neither the biokinetics of NPs, nor their subcellular distribution at different time points after administration was evaluated. It should be noted that silicon content in the liver was determined after infusion of SiO_2_NPs at a dose of 7 mg/kg in our previous work [3]. A dramatic decrease in liver silicon content was found at 20 days after infusion as compared with that observed at 1 h post-infusion. However, liver silicon content in the SiO_2_NP-treated animals was documented to be at least 6-fold higher than in the controls for up to 30 days after administration. Second, it would be important to study the dose- and size-dependent effects of both types of NPs. In the present study, we intended to perform a side-by-side comparison of SiNPs and SiO_2_NPs of approximately the same size administered at the same dose. Third, the difference obtained between SiNPs and SiO_2_NPs in histological outcomes might be attributed to the fact that a lecithin coating was used only in SiNPs, but not in SiO_2_NPs. To the best of our knowledge, phospholipids have not been previously shown to influence the rate of nanoparticle opsonization and cellular uptake. In addition, Hao *et al*. [27] recently showed that phospholipid-coated gold nanoparticles were taken up by cells much more avidly than those covered with PEG. Therefore, it is unlikely that a lecithin coating could really prevent cellular uptake of silicon nanoparticles. Lastly, the period of observation was limited to 60 days; however, it would also be helpful to study the time course of cellular response to NPs in the liver and spleen over a longer period of time, e.g., 3 to 6 months. Although the mean percentage of granulomas in RES organs was not found to be different at 5, 21, or 60 days post-infusion, the amount of granulomas in the liver and spleen tended to be greater at 60 days, especially in the SiO_2_NPs group. The possible outcomes of foreign body-type granulomas may vary from complete resolution to progression of chronic inflammation resulting in fibrosis [17]. Future studies will address these important issues.

## 3. Experimental Section

### 3.1. SiNPs and SiO_2_NPs

Silicon nanocrystals were produced in a microwave-supported plasma reactor described elsewhere [28]. Further, silicon nanocrystal powder was immersed in an aqueous solution of HF followed by gradual addition of HNO_3_ until visible photoluminescence was observed under ultraviolet illumination [29]. Etching was finished after complete consumption of HNO_3_ while the remaining HF ensured surface passivation by hydrogen. A standard fumed highly dispersed silica (Aerosil of A175 mark obtained from Vekton Ltd., Russia) was used throughout experiments.

### 3.2. Transmission Electron Microscopy

Particle sizes and morphologies of SiNP and SiO_2_NP powders were investigated by transmission electron microscopy using a Schottky field emission microscope with attached analytical equipment (JEM-2010, JEOL, Tokyo, Japan).

### 3.3. Preparation of Aqueous Suspensions of NPs

Since hydrogenated SiNPs are highly hydrophobic, the preparation of their aqueous suspension included surface coating with natural plant-derived phospholipids (lecithin, Yuwix-Pharm Ltd., Krasnodar, Russian Federation). Briefly, 100 mg of lecithin was dissolved in 10 ml of chloroform followed by the addition of 100 mg of SiNPs. The mixture was sonicated for 5 min and evaporated at 45 °C to dryness. After that, 50 mL of 0.9% sodium chloride solution were added, and the suspension of phospholipid-coated SiNPs was sonicated again for 5 min to produce a final concentration of 2 mg/mL. For preparation of aqueous suspension of SiO_2_NPs, NPs were added to 0.9% sodium chloride solution, sonicated for 5 min and left overnight. The final concentration of SiO_2_NPs was 2 mg/mL.

### 3.4. Determination of Hydrodynamic Diameter, Zeta Potential, and Surface Area of NPs

Dynamic light scattering with non-invasive back-scatter technology was used for the determination of hydrodynamic diameters of SiNPs and SiO_2_NPs. The zeta potential of both types of NPs was measured at pH = 3.5–4.0 using second-generation phase analysis light scattering Zetasizer Nano ZS device (Malvern Instruments, Malvern, UK). The surface area of the NPs was determined using the Brunauer-Emmett-Teller method.

### 3.5. Hemodynamic Measurements

Male Sprague-Dawley rats obtained from Harlan (Bicester, United Kingdom), 300–400 g in weight were used throughout the experiments. The animals were fed regular chow, and water was available *ad libitum*. All experiments were performed in accordance with the “Guide for the Care and Use of Laboratory Animals” [30] and approved by the local Ethics Committee. The animals were anesthetized with sodium pentobarbital (60 mg/kg). The trachea was intubated through a cervical incision. In spontaneously breathing animals, the right carotid artery and femoral vein were cannulated for blood pressure monitoring and anesthesia maintenance, respectively. The other femoral vein was cannulated for infusion of NPs. The volume of the first infusion of aqueous SiNP or SiO_2_NP suspensions was 0.2 mL, while for the 2 following infusions it was 0.4 mL, so that the total volume was 1 mL; the effective dose of NPs averaged 7 mg/kg. Intravenous infusions of equivalent amounts of 0.9% sodium chloride were performed in the controls. In all groups, the infusions were separated by a 20 min rest period. Heart rate (HR) and mean arterial pressure (MAP) were measured in all groups immediately before each infusion, at the end of each infusion and at 10 min following each infusion.

### 3.6. Experimental Protocol

In the major experimental series, the effects of intravenous SiNP and SiO_2_NP administration on animal body weight, hematological parameters, biochemical serum markers, organ histology and inner ear function were evaluated in the chronic treatment experiments. Male Sprague-Dawley rats weighting 300–400 g were anesthetized with sodium pentobarbital at a dose of 60 mg/kg. The left femoral vein was cannulated for infusion of NPs. The animals were randomized into the following groups: (1) vehicle-treated (n = 15) – intravenous infusion of 1 ml of 0.9% sodium chloride solution; (2) SiNPs (n = 15)—intravenous administration of SiNPs at a dose of 7 mg/kg; (3) SiO_2_NPs (n = 15)—intravenous administration of SiO_2_NPs at the same dose. The animals were left to recover after closure of the wound. On the 5th, 21st and 60th days after surgery, 5 animals from each group were sacrificed, followed by the collection of blood and tissue samples. Animal weight was determined at baseline as well as 5, 21, and 60 days after administration of NPs. Inner ear function was tested 1 day prior to surgery, and at 21 and 60 days after NP administration.

### 3.7. Evaluation of Inner Ear Function

During inner ear function testing, the animals were anesthetized by an intramuscular injection of a combination of Zoletil (0.2 mL) and Xylazine (0.02 mL). Inner ear function was evaluated by determination of distortion product otoacoustic emission (DPOAE) amplitude (NeuroAudio, Neurosoft Ltd., Ivanovo, Russian Federation) 24 h prior to the administration of NPs, as well as 21 and 60 days after NP administration. DPOAEs were elicited in response to pairs of primary tones (f1, f2), with f2/f1 approximately equal to 1.22 for all test conditions. The signal was estimated at the 2f1-f2 distortion frequency. The intensity of stimulation was L1=L2=70 dB sound pressure level (SPL). DPOAE amplitude was registered at the maximal frequencies (4.0, 5.0, and 6.4 kHz) consistent with previous observations showing that the frequencies from 4.0 to 6.0 kHz are at the lower limit of hearing in the rat.

### 3.8. Assessment of Hematological Parameters and Biochemical Serum Markers

Blood was drawn for hematology analysis using a standard saphenous vein blood collection technique. A hematological autoanalyzer (ABX Micros 60, Horiba ABX, France) was used to determine hematological parameters such as hemoglobin (HGB), red blood cells (RBC), mean corpuscular volume (MCV), mean corpuscular hemoglobin (MCH), hematocrit (HTC), mean corpuscular hemoglobin concentration (MCHC), white blood cells (WBC) and platelets (PLT). Whole blood was centrifuged twice at 3000 rpm for 10 min in order to separate serum. Using a biochemical analyzer (Cobas Integra 400 Plus, Roche Corp., Basel, Switzerland), serum biochemical analysis was carried out to determine the serum level of total bilirubin (TBIL), alkaline phosphatase (ALP), and alanine transaminase (ALT) in order to evaluate liver function. Nephrotoxicity was assessed by determination of creatinine (CREA) levels.

### 3.9. Histopathological Examination

Liver, spleen and kidney samples were fixed in buffered 10% paraformaldehyde, embedded in paraffin, cut into 5-µm sections and stained with hematoxylin and eosin (H&E) for histological examination using standard techniques. After H&E staining, the slides were observed and photos were taken using an optical microscope (DM750, Leica, Germany) at 400×. The slides were analyzed by a pathologist blinded to the treatment mode used for each group.

### 3.10. Morphometric Analysis of Liver and Spleen Sections

The density of granulomas in the liver and spleen was determined morphometrically. The granuloma area (mm^2^) and liver area (mm^2^) per H&E-stained section were determined by ImageJ2x (public domain software). The percentage of granuloma area was calculated using the formula: granuloma area per section/liver area per section × 100 [31].

### 3.11. Statistical Analysis

All data in the text are expressed as mean ± SD. Statistical analysis was performed using the SPSS 20.0 software package. The Kruskal-Wallis test was used to determine differences in the variables measured, followed by pairwise inter-group comparisons performed using a non-parametric Mann-Whitney *U* test. Differences in continuous data were tested by repeated-measures analysis of variance (ANOVA), followed by a Tukey post-hoc test. *P* values ≤0.05 were considered significant.

## 4. Conclusions

Both SiNPs and SiO_2_NPs are relatively biocompatible nanomaterials, at least when considering acute toxicity. The intravenous administration of SiNPs or SiO_2_NPs at a dose of 7 mg/kg was not associated with any changes in hematological parameters or serum biochemical markers over a period of 60 days post-infusion. Body weight and inner ear function were not different among vehicle-, SiNP-, or SiO_2_NP-treated animals. Both types of NPs were shown to cause granuloma formation in the organs of the reticulo-endothelial system, such as liver and spleen. However, SiNPs caused much less granulation in the liver and spleen then the equivalent dose of SiO_2_NPs, which should be taken into consideration during the design of novel drug delivery systems based on these carriers.
